# Atomistic simulations of graphite etching at realistic time scales†
†Electronic supplementary information (ESI) available. See DOI: 10.1039/c7sc02763j



**DOI:** 10.1039/c7sc02763j

**Published:** 2017-08-24

**Authors:** D. U. B. Aussems, K. M. Bal, T. W. Morgan, M. C. M. van de Sanden, E. C. Neyts

**Affiliations:** a DIFFER – Dutch Institute for Fundamental Energy Research , De Zaale 20 , 5612 AJ Eindhoven , The Netherlands . Email: d.aussems@differ.nl; b University of Antwerp , Department of Chemistry , PLASMANT Research Group , Universiteitsplein 1 , 2610 Antwerp , Belgium; c Eindhoven University of Technology , PO Box 513 , 5600 MB Eindhoven , The Netherlands

## Abstract

Hydrogen–graphite interactions are relevant to a wide variety of applications, ranging from astrophysics to fusion devices and nano-electronics. In order to shed light on these interactions, atomistic simulation using Molecular Dynamics (MD) has been shown to be an invaluable tool. It suffers, however, from severe time-scale limitations. In this work we apply the recently developed Collective Variable-Driven Hyperdynamics (CVHD) method to hydrogen etching of graphite for varying inter-impact times up to a realistic value of 1 ms, which corresponds to a flux of ∼10^20^ m^–2^ s^–1^. The results show that the erosion yield, hydrogen surface coverage and species distribution are significantly affected by the time between impacts. This can be explained by the higher probability of C–C bond breaking due to the prolonged exposure to thermal stress and the subsequent transition from ion- to thermal-induced etching. This latter regime of thermal-induced etching – chemical erosion – is here accessed for the first time using atomistic simulations. In conclusion, this study demonstrates that accounting for long time-scales significantly affects ion bombardment simulations and should not be neglected in a wide range of conditions, in contrast to what is typically assumed.

## Abstract 2

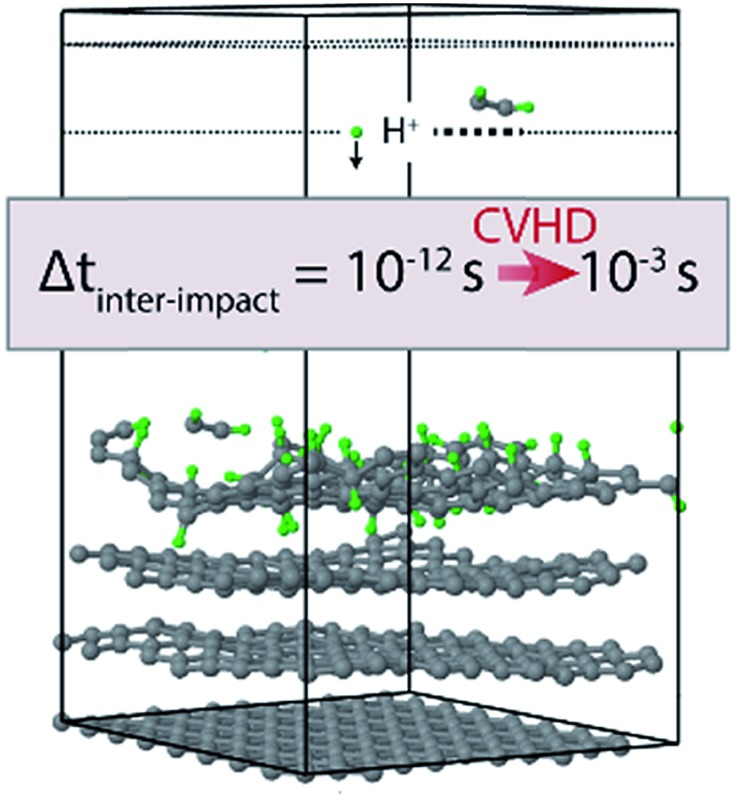
We demonstrate that long time-scale events in atomistic ion-surface bombardment simulations can be essential and need to be accounted for.

## Introduction

Fundamental hydrogen–graphite/graphene interaction has attracted interest in many research fields including astrophysics,^1^ nuclear fusion,^2–4^ fuel cells,^5,6^ gas storage,^7,8^ and nano-electronics.^9–14^ In particular, research has focused on understanding the release of hydrocarbon molecules from kinetic hydrogen ion bombardment induced chemical reactions, also referred to as chemical sputtering.^15^ A vast amount of knowledge has been gained in fusion energy research, in which dedicated experiments were performed on tokamaks^16^ and ion beam setups^17,18^ in combination with theoretical studies.^4,19–21^ Three fundamental processes were identified: physical sputtering, ion-enhanced chemical erosion, and near-surface sputtering.^15^ Using the experimental data and theoretical models as input, the correlation of these processes as functions of quantities such as the ion energy, ion flux, isotope mass, surface temperature and surface state^4,16^ was described in the semi-empirical Roth–Garcia-Rosales model.^21,22^

It remains very challenging, however, to confirm the underlying mechanisms of the above mentioned processes on the nano/micro-scale. In this regard, Molecular Dynamics (MD) simulations have been an invaluable tool, not only for the study of graphite/graphene systems as described below, but also for closely related materials such as carbon nanotubes^23–25^ and nanocrystalline diamond.^26^ In graphene/graphite etching simulations, pure graphite or pre-constructed amorphous carbon (a-C:H) samples (to which graphite samples are found to evolve)^15^ are bombarded with energetic hydrogen, thus providing insight into the elementary reactions and emission processes at the atomic level.^27–37^ For instance, Salonen *et al.* ^38^ investigated low energy H ion bombardment of a-C:H samples at 300 K, and identified a new sputtering mechanism termed swift chemical sputtering, which could provide a microscopic description of near-surface sputtering. Despiau-Pujo *et al.* ^28,39^ investigated sputtering under similar conditions, but used multi-layered graphene samples and showed that, due to the lattice structure, surface reactions and erosion become more complex and that before sputtering can occur, initial damage by hydrogenation and vacancy creation has to be induced. MD has also been employed to investigate the mechanisms behind the yield and species composition dependence on quantities such as the ion energy, surface temperature and ion flux.^38,40,41^

Unfortunately, the ion flux simulated in all of the above mentioned work exceeds the flux range of experiments by at least four orders of magnitude, which directly brings us to the general limitation of MD in terms of accessible time-scales. Typically, the time between two impacts of the etchant species on the surface is in the order of a few ps, after which it is assumed that no further events occur. Practically, in terms of chemical sputtering, this restricts MD simulations to very fast processes only, *e.g.*, physical and near-surface sputtering – both being of the order of 10 fs. Longer time-scale processes (of the order of μs to ms) that become important at elevated surface temperatures and low fluxes, *e.g.*, desorption of weakly bound species,^42^ hydrogen surface diffusion^43^ or relaxation phenomena,^44^ cannot be accessed. It is thus impossible to simulate chemical erosion, and MD studies that aim to find quantitative agreement with experiments under conditions in the range where chemical erosion is dominant have to be considered with caution.

Several methods have been proposed to improve the time-scale reach of atomistic simulations, *e.g.*, (kinetic) Monte Carlo^45–53^ and accelerated MD methods.^54–57^ From these methods, hyperdynamics is perhaps the most powerful. In hyperdynamics a bias potential (Δ*V*) is added to the global potential energy surface (PES) of the system to fill the energy minima, which reduces the waiting time between minima-to-minima transitions. The design of an appropriate bias potential for each system is, however, highly non-trivial. In the recently developed collective variable-driven hyperdynamics implementation (CVHD)^58^ the bias potential is constructed on the fly in a “self-learning” fashion, allowing the method to be more generically applicable to different systems while requiring little optimization. The flexibility of CVHD is illustrated by the wide range of processes that have already been studied with the method, including surface diffusion, conformational sampling, pyrolysis, combustion, and heterogeneous catalysis, demonstrating its ability to model complex reactions with vastly different reaction rates.^58–61^

In this work, this method (CVHD) is employed to simulate the erosion of graphite by hydrogen plasma exposure using more realistic inter-impact times (*i.e.*, up to ∼1 ms for an ion flux of ∼10^20^ m^–2^ s^–1^). As a reference, the graphite erosion is first simulated without using a bias potential. Then, the CVHD approach is adopted and the inter-impact time is varied over 9 orders of magnitude. As we will show, this has a significant effect on the ion-induced surface modification and resulting etching rate.

## Simulation model

All simulations were performed using the LAMMPS package^62^ and modified *Colvars* module.^63^ The interatomic potential used in this work is the Reactive Force field (ReaxFF).^64^ In contrast to the widely used 2^nd^ generation REBO potential,^65^ the ReaxFF potential also includes long-range van der Waals (although this was also added in ref. 28, 66 and 67) and other terms such as Coulomb and torsional interactions. In general, ReaxFF parameterizations are in good agreement with DFT results. In this work we use the parameter set developed by Mueller *et al.*,^68^ which was previously used to model hydrocarbon desorption and decomposition on Ni nanoparticles as well as CNT growth.^44^ This validates its applicability for condensed carbon nanostructures.

**Fig. 1:**
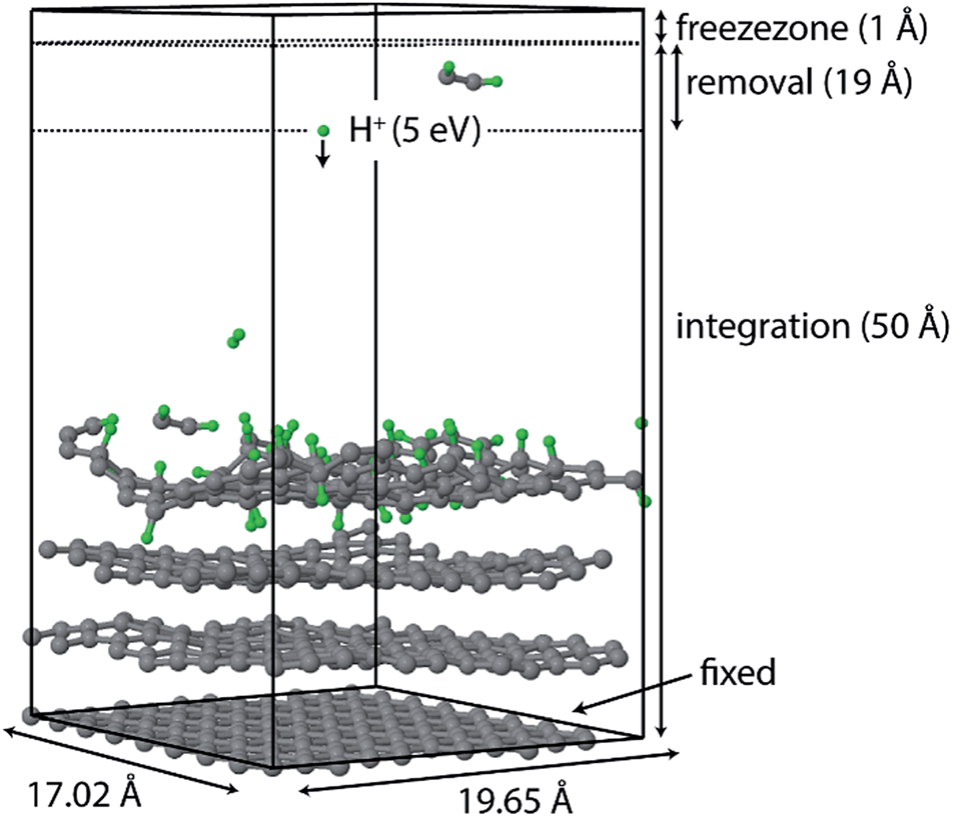
Simulation box of the erosion experiment. Hydrogen is injected at a random (*x*, *y*) position with 5 eV at *z* = 50 Å towards the graphite surface. The lower graphene layer is fixed. Eroded species are removed from the simulation once they enter the “removal zone”. The “freezezone” is introduced to prevent particle loss; in this region particles are not integrated, *i.e.*, the velocity and forces are effectively zero.

The chemical erosion of graphite by the plasma interaction was simulated by impacting the surface with 5 eV H atoms 
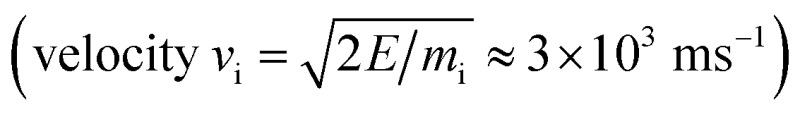
 at random (*x*, *y*) positions at normal incidence. The graphite substrate is composed of 4 graphene layers in ABAB stacking, each containing 128 carbon atoms. A sample of a simulation (after 300 H impacts) is depicted in Fig. 1. Periodic boundary conditions were imposed in the *x* and *y* directions to mimic a semi-infinite surface. Employing the Nosé–Hoover thermostat, the surface was brought to a temperature of 1000 K, which is the optimal erosion temperature of the maximum ion flux currently achieved in experiments (∼10^24^ m^–2^ s^–1^).^69,70^

After each impact, the motion of each atom was followed for 1 ps in the microcanonical (NVE) ensemble to capture the physics of the hydrogen–surface interaction (*e.g.*, reflection, adsorption or penetration). The timestep throughout the simulation was set to 0.1 fs, which was sufficiently small to conserve the total energy of the system. The integration scheme is the velocity-Verlet algorithm. After the impact of the H ion, the kinetic energy was dissipated into the material (with a typical time constant of 0.1 ps), which caused the average temperature of the substrate to rise. The natural heat conduction out of the cell is mimicked by including an additional canonical ensemble (NVT) phase for a duration of 1 ps. The substrate is cooled to its original temperature by applying a Nosé–Hoover style thermostat with a relaxation constant of 100 fs on all atoms.^71^

In previous etching simulations that were conducted using MD it would now be assumed that the system remains unperturbed during the residual time before the next impact (∼1 μs to ms) and nothing happens. In this work, however, the CVHD procedure was employed in order to reach the desired longer time-scales. In hyperdynamics, the simulated physical time, also referred to as hypertime, is obtained by multiplying the elapsed MD time by the boost factor 〈e^*β*Δ*V*^〉, in which *β* = 1/(*k*_b_ *T*) and the angle brackets denote the ensemble average, taken as the average over the whole simulation.^56^ The CVHD bias was generated by periodically adding a small repulsive Gaussian potential to the local potential energy landscape at the current state of the system. Thus the gradually increasing CVHD bias is a function of a single collective variable (CV), which includes all degrees of freedom relevant for the process(es) to be observed. A typical example of a collective variable, which was also used in this work, is based on the distortion of bond lengths from their equilibrium values (*r*_i_ – *r*_min_)/(*r*_max_ – *r*_min_). Care is taken to not add CVHD bias to the transition region, *i.e.*, the region close to the saddle point, as this would corrupt the correct sequence of events in the system, achieved here by choosing an appropriate (*i.e.*, not too large) value of *r*_max_. We used *r*_max_ = 2.2 Å and *r*_min_ = 1.5 Å for C–C bonds, which is similar to the set-up previously used for hydrocarbon pyrolysis.^59^ A combination of dynamic and static biasing was used, in which the static base level of the bias potential was set to 0.65 eV, which was found to be below the threshold for an event. On top of this static bias potential, additional Gaussian potentials of width 0.04 and height 0.01 eV were added to the PES every 100 fs, until one of the C–C bonds exceeded *r*_max_ for longer than 0.1 ps, after which the bias was removed and a new bias addition was initiated. More details about CVHD and the employed biasing method can be found in ref. 58 and 59. The C–H bond potential should in principle also be biased, but this led to a significant increase in the computation time. Bearing in mind the aim of the current work – to qualitatively show the effect of long time-scales on the physics and chemistry of the simulated system – C–H bond potential biasing will be left for future work.

In contrast to most standard hyperdynamics simulations, no “fixed” time-scale elongation is obtained. That is, the total magnitude of the bias potentials (and hence the overall time-scale that can be reached) is not a fixed quantity, but dependent on the requirements of the system. In addition, each CVHD cycle only lasts as long as needed to reach a certain inter-impact time. Owing to this flexible biasing strategy, arbitrarily long time-scales can be simulated, allowing us to model different ion flux regimes over several orders of magnitude. The typical calculation performance for the simulation with the longest inter-impact interval of 1 ms is ∼1 h wall time (for parallel operation on 4 CPU cores of an Intel® Core i7-2600K, 3.4 GHz, 8 MB cache). From this wall time only up to 66% was spent on CVHD. After each CVHD phase a new impact was initiated (*i.e.*, starting from the NVE phase).

Once erosion was initiated and eroded species entered the removal zone, they were deleted from the simulation after each simulation phase (*i.e.*, NVE, NVT, and CVHD). An additional zone was applied to prevent species from leaving the simulation box; in this zone the atoms were not integrated.

## Results

### Short time-scale simulation

**Fig. 2:**
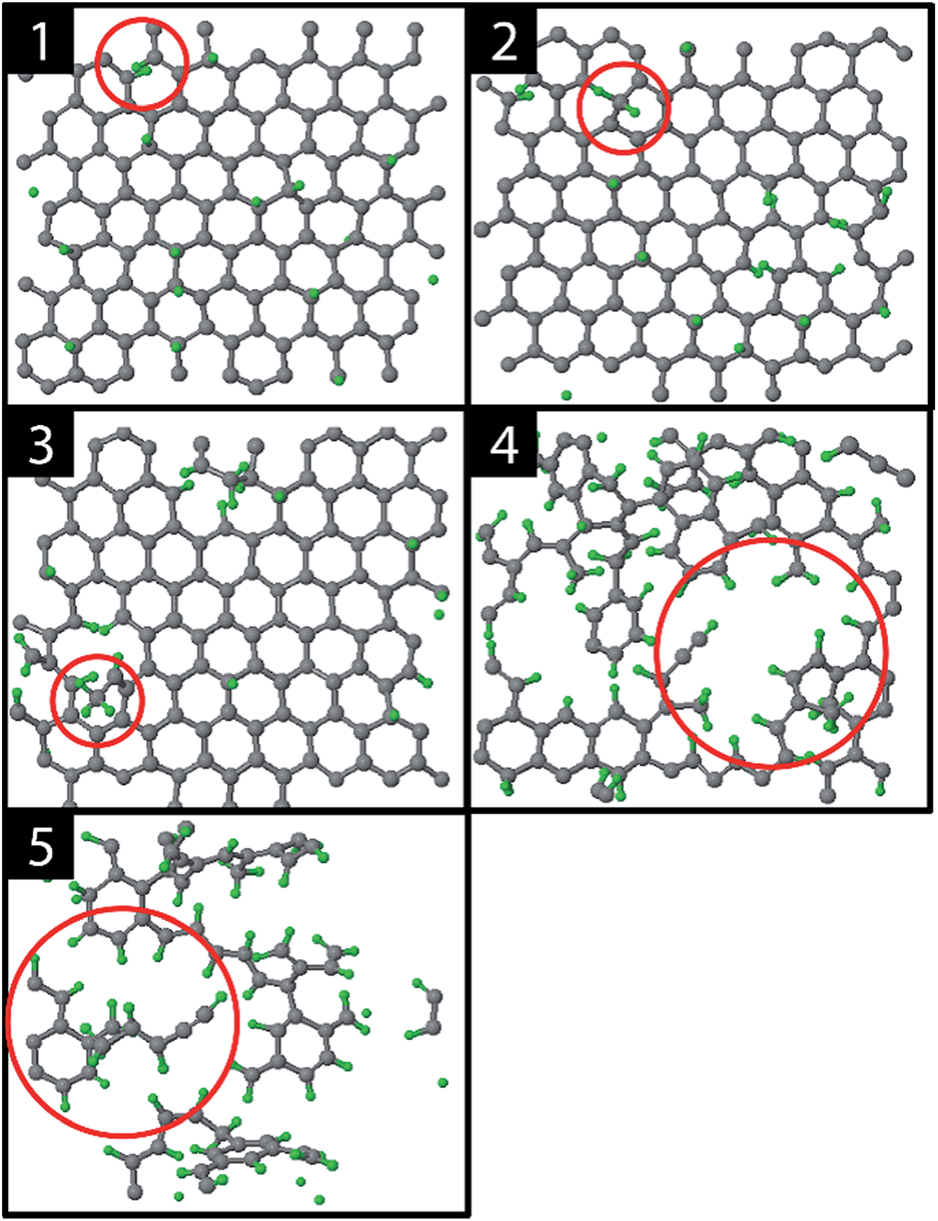
Several stages during erosion of the top layer of the graphite substrate: (1) hydrogenation of the top layer, (2) the first CH_2_ fragment (red circle), (3) the first CH_3_ fragment (red circle), (4) holes and (5) carbon islands.

The graphite erosion was first simulated with an inter-impact time of only 3 ps, from which 1 ps CVHD phase. Fig. 2 shows the time evolution of the top layer of the graphite sample after an increasing number of impacts. From the impacting H ions, a fraction reflected from the surface (mainly from the hollow and bridge sites). Another fraction was chemisorbed on the surface at C–C dimer locations after sp^2^/sp^3^ re-hybridization (Fig. 2; 1). This is a similar behavior to that in ref. 28 and is expected because the interatomic potential curves are similarly shaped (for a DFT comparison of the interatomic potential curves near pristine graphene, see Fig. S1†). In contrast to ref. 28, however, some H atoms were able to penetrate the top layer and become adsorbed on its back side, *e.g.*, through the bridge site, which may be related to the reduced energy barrier. The chemisorption of the H atoms displays a preference for *ortho*- and *para*-pairs – also referred to as clustering, which can be explained by the increased binding energy of these pairs as compared to two separately bound H atoms.^39,72,73^

The hydrogenation process eventually led to bond breaking due to mechanical stress, either induced by local impacting H ions or by repulsion of a hydrogen pair in the *ortho* configuration (encircled in Fig. 2; 1). The two unsaturated C atoms that were formed after the C–C bond breaking rapidly saturated their dangling bonds by forming CH_2_ and CH_3_ groups, as depicted in Fig. 2; 2–3 (for a DFT comparison of the interatomic potential curves near a defected graphene see Fig. S3†). Subsequently, additional H atoms emerged at the back side of the top layer because of surface restructuring. Once the CH_2_ and CH_3_ groups were formed, carbon was etched by volatile product formation, as further explained below. With increasing fluence, the C–C rupturing and etching led to holes in the graphene layer (Fig. 2; 4) and eventually to the formation of islands of hydrogenated carbon atoms (Fig. 2; 5), which were etched rapidly. Once holes – so-called etch pits – were formed, the 5 eV H ions could undisturbedly penetrate the active layer and start hydrogenating the second layer. This vertical ‘graphite peeling’ process continued layer by layer, consistent with ref. 28, 30 and 37.

**Fig. 3:**
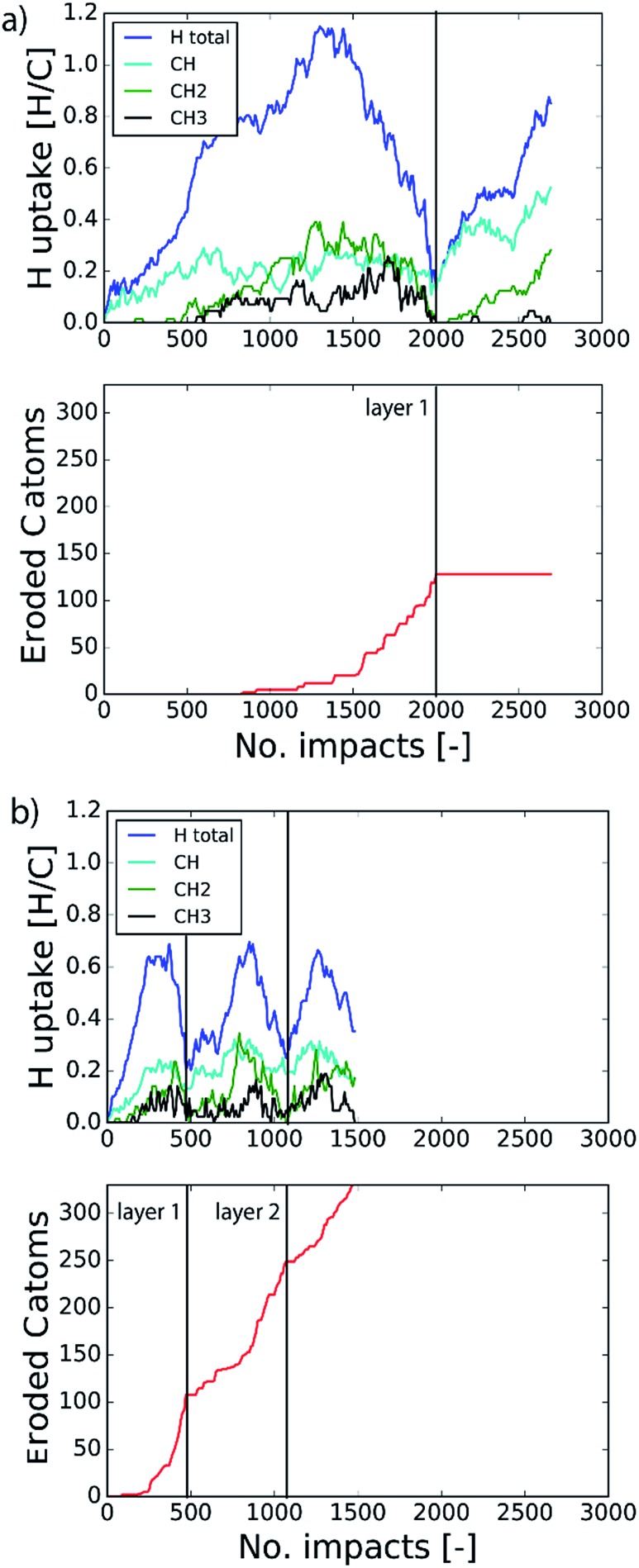
Time evolution of (top) the total hydrogen uptake in the system and the present hydrogen in the CH, CH_2_ and CH_3_ groups, and (bottom) the eroded number of carbon atoms as a function of the number of impacts. The inter-impact time interval is (a) 3 ps and (b) 1 ms.

The etching process was investigated more systematically by monitoring the hydrocarbon groups on the surface. In Fig. 3a the hydrogen uptake in the system is shown as a function of the number of impacts as well as the contribution of hydrogen in the CH, CH_2_, and CH_3_ groups. It shows that the surface was quickly hydrogenated within 500 impacts and then saturated to a CH/C ratio of ∼40% in the first layer, which is in the range of hard a-C:H films.^74^ This saturation value can partly be explained by the curvature of the graphene layer, which distorts the sp^2^ and sp^3^ hybridization states due to mechanical stress.^5^ At valley locations this reduces the binding energy and hence incoming H ions are more easily reflected. Once the surface was sufficiently hydrogenated, CH_2_ groups appeared which led to an increased H uptake in the first layer. After ∼600 impacts CH_3_ groups were also observed and finally after ∼800 impacts, etching was initiated. This is in line with the chemical erosion model of ref. 19, 21 and 22, in which hydrogenation leads to sp^3^ complexes, C–C bond breaking and eventually formation of hydrocarbon complexes at the surface, *e.g.*, CH_3_ radicals. Finally, the loosely bound hydrocarbon groups were etched, as further explained below. Etching continued until all of the carbon and hydrogen atoms in the first layer were released (at ∼2000 impacts). Hereafter, the same cycle was re-initiated.

**Fig. 4:**
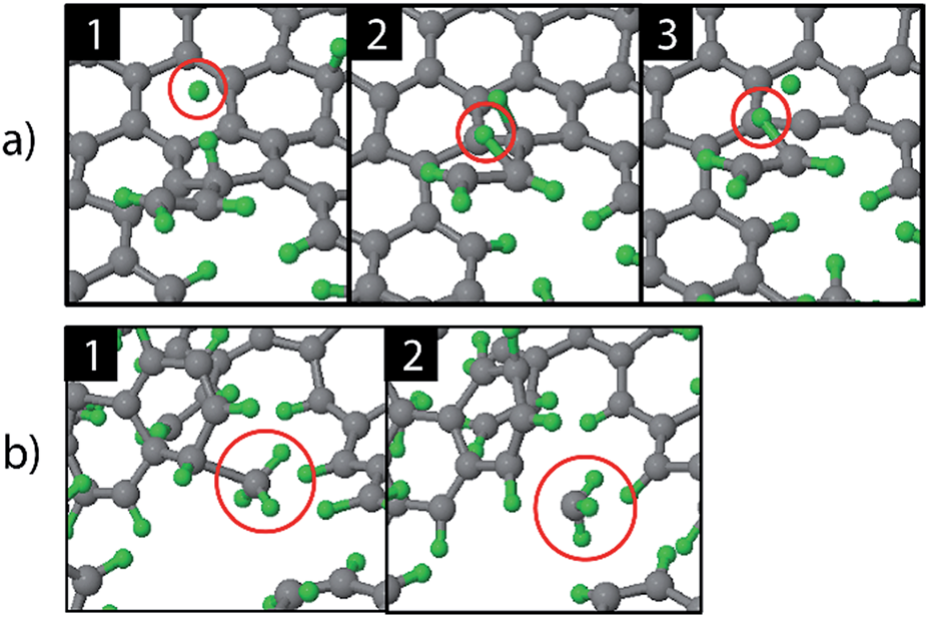
Observed erosion mechanisms: (a) hydrogen ion impact induced erosion (the incoming H ion is encircled in window (1), and (b) CH_3_ erosion due to a thermal fluctuation.

The etching mechanisms were examined in more detail by observing the exact moment of etch product release. Two possible paths were identified as depicted in Fig. 4. In the first case, an ion impacted very close to a carbon chain (Fig. 4a), eventually causing the release of a hydrocarbon molecule (C_2_H_2_ in this case). The release of a weakly bound hydrocarbon molecule due to an ion impact is hereafter referred to as ion-induced erosion. This process points towards swift chemical sputtering as described in ref. 38. In this case, the ions can directly break the covalent C–C bonds of surface hydrocarbon groups bound to the carbon network, because the carbon atoms are forced apart due to the repulsive part of the potential energy function.^38^ Since this repulsion occurs very quickly, the surrounding carbon network has no time to relax.

In the second case, a loosely bound CH_3_ group was desorbed in a thermal fluctuation (Fig. 4b). The release of a weakly bound hydrocarbon molecule due to a thermal fluctuation is hereafter referred to as thermal-induced erosion. The observed mechanism is similar to the ion-enhanced chemical erosion process that was extensively studied in ref. 19, 21 and 22. In these reports, the erosion mechanism is thought to proceed by the following steps. After significant hydrogenation of the surface, a fraction of the carbon atoms will be in an intermediate radical state sp^*x*^ (with free/dangling bonds) which is either caused by the hydrogenation of an sp^2^ C radical or due to abstraction of a bound H atom on an sp^3^ C atom by an Eley–Rideal type process. At elevated temperature (>400 K), a C atom that contains a hydrocarbon group (*e.g.*, CH_3_) and neighbors such an sp^*x*^ C radical can release this hydrocarbon group; the dangling bond of the C atom will form a double bond with the neighboring free bond of the sp^*x*^ C radical (also called β-scission). Alternatively, the hydrocarbon group can directly thermally desorb after the hydrogen irradiation is stopped.^22^ Remarkably, our simulation shows that this latter process may also occur during hydrogen irradiation, because no hydrogen abstraction was observed before the hydrocarbon complex release.

**Fig. 5:**
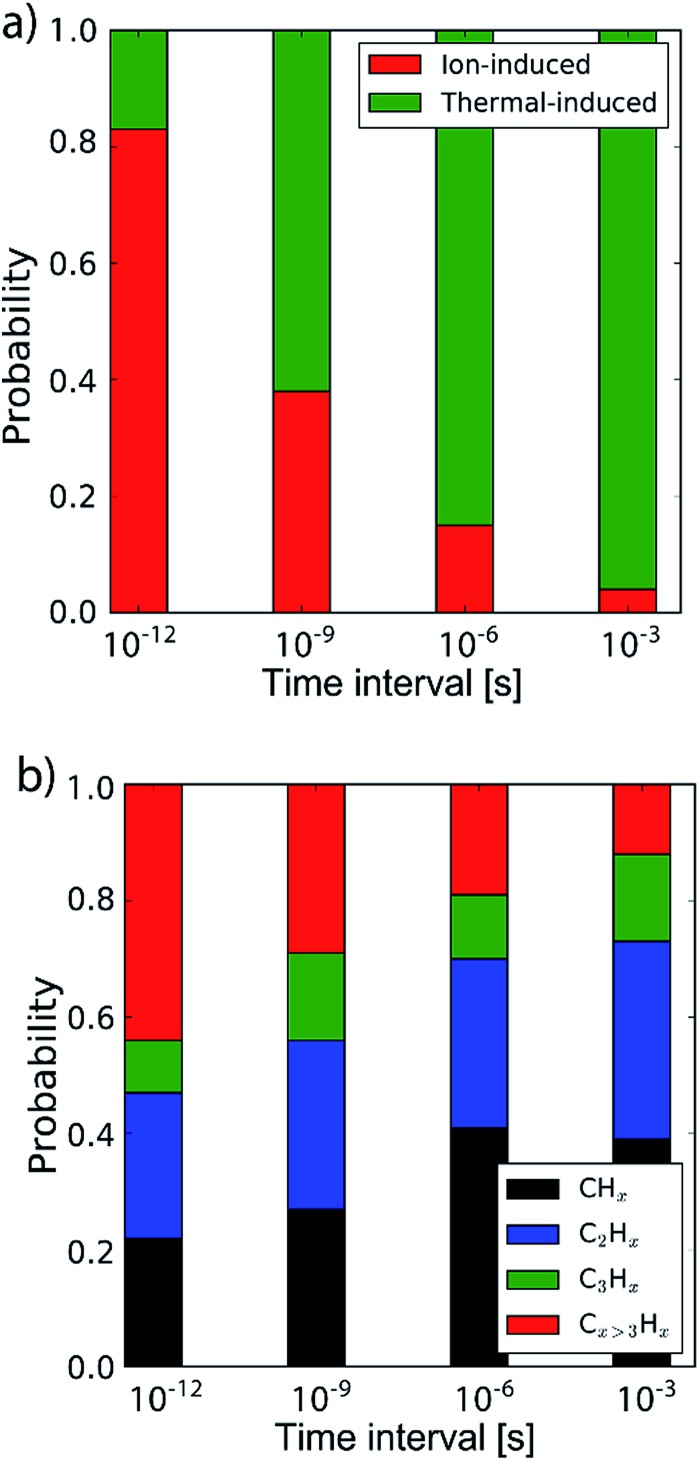
(a) The probability of the erosion mechanism type (ion- or thermal-induced), for varying inter-impact times: 3 ps, 1 ns, 1 μs and 1 ms. (b) The distribution of the erosion species.

By determining the exact point at which the hydrocarbon molecule was disconnected from the carbon network, the probability of ion- or thermal-induced release was estimated, as depicted in Fig. 5a. The results show that over 90% of the release is ion-induced.

Lastly, the erosion species distribution is depicted in Fig. 5b. Surprisingly, the distribution consists of a significant fraction of large hydrocarbons (C_*x*>3_H_*y*_) compared to the literature.^27,30^ These large hydrocarbons mainly originate from the carbon islands observed in Fig. 2; 5. Due to the weak van der Waals binding, these groups are only loosely bound to the surface and thus could be desorbed, enhanced by energy transferred from the incoming H ions. These carbon islands predominantly comprise the fraction of hydrocarbons with five or more carbon atoms.

### Long time-scale simulation by C–C bond biasing

**Fig. 6:**
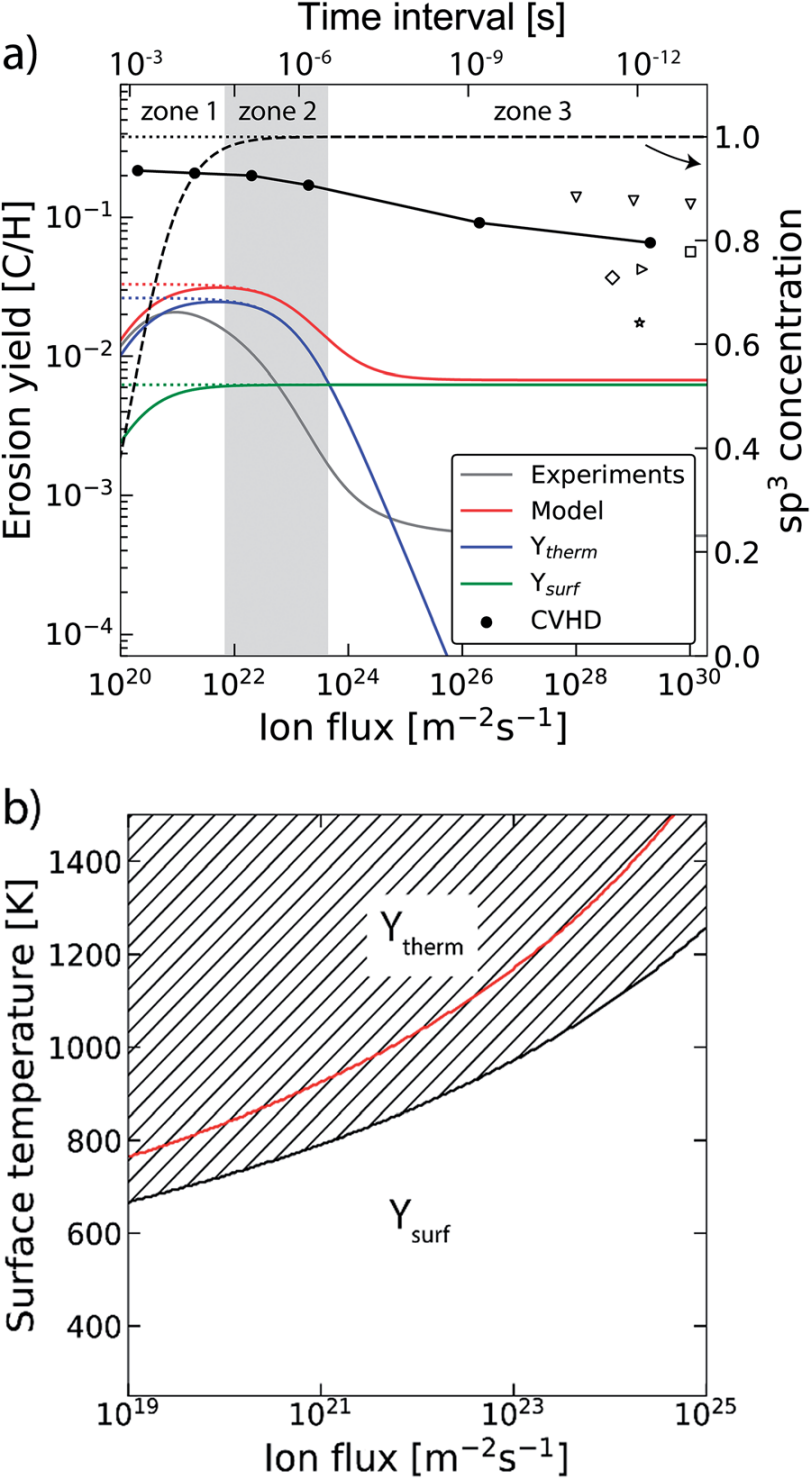
(a) The erosion yield of graphite at 1000 K using our CVHD simulation (). For comparison, the data from previous MD simulations of H ion bombardment on: a-C:H at 1000 K (▽ (ref. 35)), a-C:H at 300 K (□ (ref. 35), (ref. 27) and (ref. 76)) and graphite at 300 K (☆ (ref. 39)). Moreover, the calculated erosion yield using the Roth–Garcia-Rosales model is depicted as a function of the ion flux based on the unmodified model. The contributions of the thermal (*Y*_therm_), near-surface erosion (*Y*_surf_), and sp^3^ concentration (dashed curve, right-axis as indicated by the arrow) are plotted separately. Lastly, the erosion yield and sp^3^ concentration are depicted in the case of negligible desorption (dotted curves). (b) The transitional surface temperature from ion- to thermal-induced erosion as a function of the flux (black curve), where the marked area is the regime of dominant thermal-induced erosion. The red curve shows the maximum erosion temperature.

In the previous section the time between impacts was set to 3 ps. In this section, we apply CVHD to investigate the effect of moving towards more realistic time intervals, *e.g.*, 1 ms, corresponding to an ion flux of 10^20^ m^–2^ s^–1^. In Fig. 3b the time evolution of hydrogen uptake and erosion is plotted for 1 ms inter-impact time intervals. It appears that for all of the time intervals, the erosion process can be described by the same steps as those described in the previous section: hydrogenation, CH_2_/CH_3_ group formation, and etching of the top graphene layer. The maximum total H surface coverage – defined as the ratio of the total number of H atoms to the number of C atoms in the top graphene layer – appears to decrease with increasing inter-impact time (from ∼110% to ∼70% H/C) while the relative fraction of CH_3_ groups increases (from ∼20% to ∼30%). More striking, however, is that the erosion stages appear to occur earlier with increasing inter-impact time. The number of impacts before the H uptake reaches 30% is reduced from ∼370 to ∼150, while erosion starts after ∼80 impacts instead of ∼850 as was the case in the short time-scale simulation. This is also consistent with Fig. 6a, in which the erosion yield (number of eroded carbon atoms per incoming ion) is depicted as a function of the inter-impact time (and ion flux). In the studied range, the erosion yield shows a monotonic increase of a factor of 3.3. For inter-impact times of >10 μs the erosion yield appears to increase more slowly. The erosion yield in the case of a 3 ps inter-impact time (0.07 C/H) is a factor of four higher compared to ref. 28, which is probably related to the lower surface temperature (300 K) in that simulation.

The erosion species distribution for varying inter-impact times is depicted in Fig. 5b. The CH_*x*_ and C_2_H_*x*_ contributions appear to increase with increasing inter-impact time, while the contribution of large hydrocarbon molecules (C_*x*>3_H_*y*_) decreases. Because the majority of the large molecules are released as carbon islands, this suggests that these clusters disintegrate before they can desorb due to the erosion of small hydrocarbon molecules, *i.e.*, C_*x*=1–3_H_*y*_. With regard to the smaller hydrocarbon molecules, the contribution of CH_*x*_ is initially lower than that of C_2_H_*x*_, but it starts to dominate the composition for inter-impact times above 1 ns. Moreover, Fig. 5a shows that between 3 ps and 1 ns a transition occurs from ion- to thermal-induced erosion. In the case of 1 ms inter-impact time, ∼95% of the eroded particles are thermal-induced.

## Discussion

The erosion process is affected in several ways by the long time-scales between impacts. While similar steps appear to be followed as in the short time-scale simulation, these steps are observed to occur earlier in time, *i.e.*, after fewer impacts. This can be understood by considering the biasing acceleration method which has been applied. The biasing effectively brings the C–C bond length close to the value before bond breaking occurs, which simulates the local mechanical stress associated with thermal fluctuations in the graphene surface. With increasing inter-impact time, the effect of these fluctuations will be more pronounced, *i.e.*, for the same number of impacts, C–C bonds are more likely to break (note that this only applies however for the C–C bonds that have an already reduced bond energy, *e.g.*, by the presence of H atoms). The subsequent rise in the number of broken C–C bonds leads to more dangling bonds at the surface, and hence to a higher probability for H ion adsorption. Consequently, saturation is reached earlier while CH_2_/CH_3_ group formation is promoted. Additionally, the desorption of small hydrocarbon molecules (C_*x*=1–3_H_*y*_) by thermal fluctuations is enhanced. All these effects lead to a boost in the erosion yield. For inter-impact times exceeding 10 μs, however, the yield appears to increase more slowly. This may be explained by the depletion of the number of potential bond breaking events due to surface relaxation.

Concerning the species distribution, a shift is observed from C_2_H_*x*_ to CH_*x*_ release with increasing inter-impact time, which may be explained by the transition from ion- to thermal-induced erosion. Moreover, in contrast to other MD work,^35,38,40,75^ a significant fraction of large hydrocarbon molecules C_*x*>4_H_*x*_ is observed in the case of short inter-impact times (3 ps and 1 ns), which we attribute to the release of the carbon islands bound by van der Waals forces. The discrepancy with the literature may be explained by the difference in the sample structure. In the simulations of ref. 35, 38, 40 and 75 an amorphous carbon sample was used instead of graphite, thus carbon islands are not likely to form. Nonetheless, the release of carbon islands may have been boosted due to the shape of the interatomic potential selected in this work. This could result in an overestimation of the erosion yield by less than 60% in the case of a 3 ps inter-impact time *Δ*.

The trend of a rising erosion yield and the shift towards thermal-induced erosion as a function of the increasing inter-impact time can be explained by the semi-empirical Roth–Garcia-Rosales model.^3^ In this model the total chemical sputtering yield is expressed as:

1 *Y*_tot_ = *Y*_phys_ + *Y*_therm_(1 + *DY*_dam_) + *Y*_surf_,

where *Y*_phys_, *Y*_therm_, and *Y*_surf_ are the erosion yields due to physical sputtering, chemical erosion and near-surface processes, respectively, and *DY*_dam_ is an additional multiplicative term that includes a radiation damage yield *Y*_dam_ and material isotope dependent constant *D*. Fig. 6a shows the erosion yield calculated by this model for 5 eV ions impacting graphite at a surface temperature (*T*_s_) of 1000 K as a function of the ion flux (*φ*), hereby adopting the equations and parameters of ref. 3. The results are plotted with and without including the empirically obtained flux effect compensation factor (*C* ∝ *φ* ^–0.54^). To gain more insight, the contributions of the thermal and surface erosion (without flux effect) are plotted separately. Note that beyond an ion flux of *Γ*_max_ = 6 × 10^23^ m^–2^ s^–1^ no experimental data are available and the trend is unknown. The results of the current CVHD study are included along with (for comparison) the data of MD studies in the literature, although different sample structures and temperatures were used. The yields of the literature data were scaled to 5 eV using the ion energy dependence of ref. 70. Three zones can be distinguished, dominated by: (1) desorption, (2) chemical erosion and (3) near-surface-chemical sputtering. Apart from the large deviation between the absolute values of the erosion yields obtained by CVHD and the model, they show qualitatively the same trend in terms of the yield. Additionally, Fig. 5a shows that the transition from ion- to thermal-induced erosion is reproduced. The order of magnitude overestimation of the yield by the CVHD simulation compared to the semi-analytical model can be explained by the difference in rate constants and thus by the precise shape of the interatomic potential, but can also be related to factors which are not considered in the current work, such as the microscopic morphology. Furthermore, the results suggest that the empirically obtained trend of rapidly decreasing erosion yield as a function of the flux (∝*φ* ^–0.54^) is not observed in our atomistic simulations, at least beyond *Γ*_max_, *i.e.*, the data fitting range. In line with ref. 35, this suggests that this effect may not be related to the properties inherent to the material, but is most likely caused by external factors, *e.g.*, redeposition of eroded hydrocarbon molecules or processes occurring in the plasma.

The transition from ion- to thermal-induced sputtering is visualized in Fig. 6b as a function of the surface temperature and ion flux (calculated by the aforementioned model). The marked area shows the regime of dominant thermal-induced erosion, which is inaccessible by common MD simulations. In particular for low flux simulations (*Γ*_max_ ∼ 10^19^ m^–2^ s^–1^), this regime is already entered for *T*_s_ < 670 K. Thus, MD simulation studies on atomic processes in this range have to be considered with caution.

In our simulations C–H bond biasing was not included. Possibly, this led to an underestimation of processes such as thermal hydrogen desorption and hydrogen surface diffusion followed by Langmuir–Hinshelwood recombination. Based on the Roth–Garcia-Rosales model it is expected that neglecting such desorption processes only leads to a saturation of the yield for fluxes below 10^22^ m^–2^ s^–1^ (black dotted line in Fig. 6a), instead of a rapid drop (black dashed line). This trend is consistent with our CVHD results. Nevertheless, unforeseen effects may be significant.

## Conclusion

This work presents the effect of long time-scales on graphite erosion by hydrogen ion bombardment using a recently developed hyperdynamics implementation. The results show that while the types of graphite erosion process – hydrogenation, vacancy creation and volatile product formation – do not depend on the inter-impact time, a clear reduction of the required fluence was observed with increasing the inter-impact time. Moreover, the increase in inter-impact time resulted in an increased erosion yield, a reduction of the maximum hydrogen surface coverage and a shift towards smaller hydrocarbon species release. This could be explained by the higher probability for C–C bond breaking due to the prolonged exposure to thermal stress and the associated transition from ion- to thermal-induced etching. In fact, this latter process – chemical erosion – could be accessed for the first time by atomistic simulations due to extended time-scales and is supported by semi-empirical modelling. In conclusion, this study demonstrates that long time-scales can have several important effects on ion bombardment simulations and in contrast to what is typically assumed in the literature these effects may not be neglected, especially for low flux ion bombardment simulations.

## Conflicts of interest

There are no conflicts to declare.
