# Supplementary material for: Atomistic simulations of graphite etching at realistic time scales
Source: Chem Sci. 2017 Aug 24;8(10):7160–8. doi: 10.1039/c7sc02763j (PMC5635421; doi:10.1039/c7sc02763j)
Supplement: Supplementary file 1 [file SC-008-C7SC02763J-s001.pdf]

Electronic Supplementary Material (ESI) for Chemical Science.
This journal is © The Royal Society of Chemistry 2017

Supplementary Information

Atomistic simulations of graphite etching at realistic time scales

D. U.B. Aussems[a], K.M. Bal[b], T. W. Morgan[a], M. C. M. van de Sanden[a,c] and E. C. Neyts[b]

[a.] *DIFFER - Dutch Institute for Fundamental Energy Research, De Zaale 20, 5612 AJ Eindhoven, the Netherlands.*
[b.] *University of Antwerp, Department of Chemistry, PLASMANT Research Group, Universiteitsplein 1, 2610 Antwerp, Belgium.*
[c.] *Eindhoven University of Technology, PO Box 513, 5600 MB Eindhoven, The Netherlands.*

Fundamental interactions of H atoms impinging on pristine graphene (classical MD vs DFT)

In order to determine the ion-surface interaction dynamics we let a 25 eV H atom imping on the top, bridge and hollow sites of a graphite sample at 0 K. The potential energy difference of this H-graphene system and the same system at infinite separation is depicted in Fig. S1a,b as function of the distance to the surface (defined as the difference in z-location of the impacting H atom and the nearest C atom). Before the H atom is deposited the energy of the system is minimized by allowing it to expand, and it was further relaxed by integrating it for 0.4 ps in the NVE phase. The results are compared to the results of the DFT calculations described in Ref. [1]. In these calculations, the surface is kept fixed while the H atom was moved towards the surface. The results were obtained using a GGA functional and it includes spin polarization. For further details we refer to the paper. Note that in contrast to Ref. [1], here we applied the REAX-FF[2] with the parameter set of Mueller et al.[3] The results shows that at the bridge site, an energy barrier exists (10.7 eV), which is lower than DFT (17.4). This may be related to the fixed positions of the C atoms in DFT. Moreover, a similar plateau is observed around 1-1.5 Å, although the magnitude in (classical) MD (4 eV) and DFT (0.5 eV) deviate. In the case of the top site, a metastable chemisorption site can be observed around the local minimum at 1.5 Å and with an energy barrier of 0.9 eV. This is 0.39 eV in DFT (and at 1 Å). The agreement of the potential well position is much better predicted for lower energy H ions (1.1 Å for 1.3 eV impact energy, see Fig. S2). This is related to the longer interaction time, which allows for sp2-sp3 re-hybridization. Finally, at the hollow site a similarly shaped energy barrier exists (7.7 eV) which is lower in DFT (3.3 eV). To conclude, the predictions of MD and DFT are qualitatively (in shape) comparable, although the quantitative deviations.

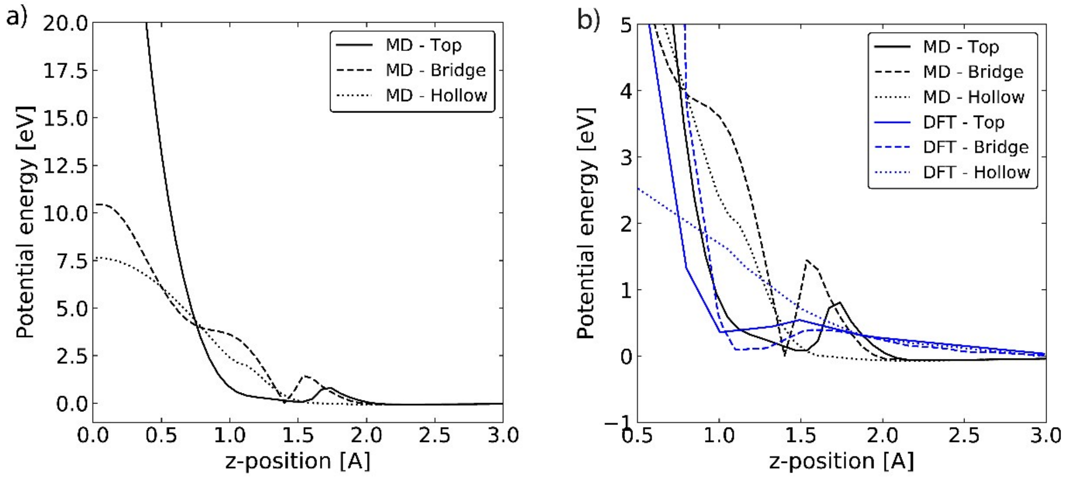

Fig. S1: a) The potential energy curves of hydrogen impact on the top, bridge and hollow sites of graphene. b) A zoom-in on the results and display of the DFT results from Ref. [4].

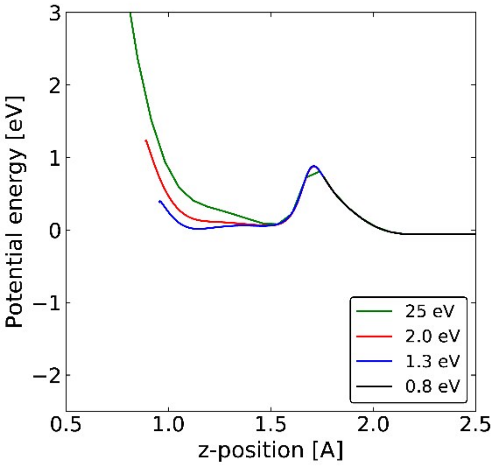

Fig. S2: a) The potential energy curves of a kinetic H atom impacting on the top site of graphene at 0 K for varying impact energies.

Fundamental interactions of H atoms impinging on defected graphene (classical MD vs DFT)

Graphite etching can eventually lead to defect formation in the graphene surface, which can influence the interaction of kinetic hydrogen ions with the surface. Here, we solely limit the discussion on a central mono-vacancy and compare our MD simulation results with DFT calculation performed in Ref. [1]. The same methodology is applied as the section above. The potential energy difference of the H-graphene system and the same system at infinite separation is depicted in Fig. S3. The results show that the vacant atomic site (top) is purely attractive, which is consistent with DFT, although the potential energy starts to drop at a shorter distance to the surface (around 1 Å instead of 2 A in DFT). Moreover, a similarly shaped potential well is predicted, which has a depth of 4 eV instead of 2.1 eV in DFT. Lastly, the value for the energy barrier of the hollow site (2 eV) agrees well. So, also in the case of damaged/defected graphene a satisfactory qualitative agreement is found between MD and DFT.

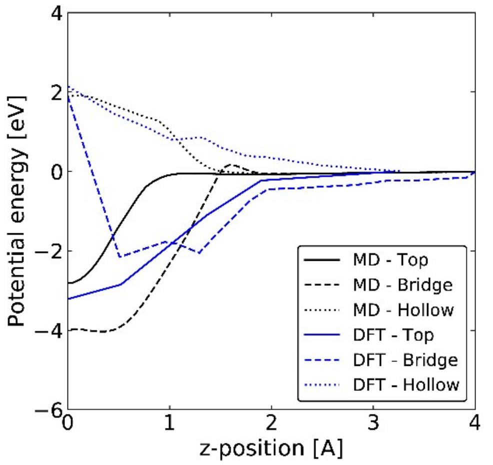

Fig. S3: The potential energy curves of a H atom impinging on the top, bridge and hollow sites of defected graphene at 0 K. DFT values are from Ref. [4].

References

1 E. Despiau-Pujo, A. Davydova, G. Cunge, L. Delfour, L. Magaud and D. B. Graves, *J. Appl. Phys.*, 2013, **113**, 114302.
2 A. C. T. van Duin, S. Dasgupta, F. Lorant and G. W. A., *J. Phys. Chem. A*, 2001, **105**, 9396–9409.
3 J. E. Mueller, A. C. T. Van Duin and W. A. Goddard, *J. Phys. Chem. C*, 2010, **114**, 5675–5685.
4 E. Despiau-Pujo, A. Davydova, G. Cunge and D. B. Graves, *Plasma Chem. Plasma Process.*, 2016, **36**, 213–229.
